# Evaluating the Role of Ultrasonographic Measurements in Assessing Macrosomia Among the Fetuses of High-Risk Antenatal Women in a Tertiary Care Hospital

**DOI:** 10.7759/cureus.92882

**Published:** 2025-09-21

**Authors:** Rajeswari I., Nirupa S., Pavithra M.

**Affiliations:** 1 Obstetrics and Gynaecology, Sree Balaji Medical College and Hospital, Chennai, IND

**Keywords:** estimated fetal weight (efw), fetal macrosomia, gestational diabetes mellitus (gdm), high-risk pregnancy, obstetrics ultrasonography

## Abstract

Background and objective

Fetal macrosomia, defined as a birth weight >4.0 kg, is associated with serious maternal and neonatal complications such as prolonged labor, cesarean delivery, postpartum hemorrhage, birth trauma, and long-term metabolic risks. Maternal factors, particularly gestational diabetes mellitus (GDM), excessive weight gain, and certain comorbidities, increase the risk of macrosomia. Early detection in high-risk pregnancies is crucial to guide clinical management and improve outcomes. Conventional sonographic estimated fetal weight (EFW) provides important information but may lack accuracy. Novel markers such as umbilical cord thickness (UCT), fetal fat layer (FFL), and shoulder pad thickness (SPT) have shown potential predictive value. This study aimed to evaluate the accuracy of these sonographic parameters in predicting fetal macrosomia among high-risk pregnancies in a tertiary care setting.

Methodology

This prospective hospital-based study was conducted at Sree Balaji Medical College and Hospital, Chennai, from July 2023 to June 2024. A total of 100 high-risk antenatal women at 35-36 weeks of gestation were recruited through purposive sampling. Inclusion criteria were pregnancies complicated by GDM, anemia, hypertensive disorders, bronchial asthma, epilepsy, or cardiac conditions. Women with multiple pregnancies, fetal anomalies, or unwillingness to participate were excluded. After informed consent, detailed histories and anthropometric data were recorded, followed by ultrasonographic measurements of UCT, FFL, SPT, and EFW. Outcomes were followed up till delivery.

Results

Out of the total 100 participants, the majority of women were aged 31-35 years (44%) and primigravida (59%). High-risk conditions included GDM (65%), anemia (16%), and hypertensive disorders (9%). Macrosomia occurred in 49% cases, predominantly among overweight women (37/49, 75.5%), upper socioeconomic class (45/49, 92.3%), and GDM mothers (49/65, 75.4%) (all p<0.001). Male infants were more affected (28/49, 57.1%, p=0.034). UCT >90th percentile, FFL >5 mm, and SPT >12 mm were exclusively associated with macrosomia (all p<0.001). EFW >4.0 kg predicted macrosomia with 100% accuracy. Macrosomia was most frequent in *LSCS *deliveries (19/20, 95%).

Conclusion

Umbilical cord thickness, fetal fat layer, and shoulder pad thickness are reliable predictors of fetal macrosomia in high-risk pregnancies. GDM emerged as the strongest maternal risk factor. Incorporating these sonographic markers into routine third-trimester screening may improve early detection, optimize delivery planning, and reduce maternal and neonatal complications.

## Introduction

Fetal macrosomia, commonly defined as a birth weight exceeding 4,000 grams or above the 90th percentile for gestational age, poses a significant challenge in modern obstetric practice [1]. Accurate prenatal detection of macrosomia has gained increasing importance due to its strong association with adverse maternal and neonatal outcomes. In the setting of high-risk pregnancies, particularly those managed in tertiary care facilities, the role of ultrasonographic assessment in predicting macrosomia is critical for timely clinical decision-making and optimizing maternal-fetal outcomes. Globally, macrosomia occurs in approximately 3-15% of all pregnancies [2]. In developed countries, its prevalence ranges from 5% to 20% of all births, with an observed increase of about 15-25% over the past two decades [3].

Maternal diabetes remains the leading risk factor for fetal macrosomia. In particular, gestational diabetes mellitus (GDM) has profound implications on fetal growth patterns through increased maternal-fetal glucose transfer across the placenta. This hyperglycemic intrauterine environment stimulates excessive fetal insulin production, which in turn promotes accelerated somatic growth and increased fat deposition [4]. The public health relevance of this issue is underscored in India, where the prevalence of diabetes is projected to rise dramatically from 31.7 million in 2000 to 79.4 million by 2030 [5]. Such trends highlight the urgent need for effective strategies to detect and manage fetal overgrowth in diabetic pregnancies.

In addition to diabetes, excessive maternal weight gain during pregnancy represents another important and modifiable determinant of macrosomia [6]. Gestational weight gain exerts a direct influence on fetal growth by enhancing nutrient supply, thereby predisposing to excessive fetal size when uncontrolled. Obstetric history also contributes to risk, as multiparous women demonstrate a higher likelihood of delivering macrosomic infants compared to primigravidae [7]. Furthermore, fetal sex has been shown to influence birth weight, with male fetuses typically weighing more than females.

The complications associated with fetal macrosomia are diverse and clinically significant. Labor complications are a primary concern, with shoulder dystocia being one of the most dangerous obstetric emergencies. This condition requires prompt recognition and skilled manoeuvres for resolution, as delayed management can result in brachial plexus injuries, neonatal asphyxia, or even perinatal death, in addition to maternal morbidity [8]. Other maternal risks include genital tract lacerations, particularly severe third- and fourth-degree perineal tears, which carry long-term sequelae such as pelvic floor dysfunction and fecal incontinence. Operative vaginal deliveries, which may become necessary in macrosomic births, further amplify these risks.

Postpartum hemorrhage is another important complication, often resulting from uterine overdistension and atony following the delivery of a large fetus. In women with a scarred uterus, macrosomia significantly increases the risk of uterine rupture, a catastrophic event that necessitates urgent surgical intervention [9]. From the neonatal perspective, macrosomia is associated with both immediate and long-term health consequences. Neonatal hypoglycemia, particularly in infants of diabetic mothers, arises from persistently high fetal insulin levels after the abrupt withdrawal of maternal glucose at birth. In the long term, macrosomic infants face an elevated risk of obesity, insulin resistance, and metabolic syndrome during childhood and adulthood [10].

Given the spectrum of risks associated with macrosomia, early identification of at-risk pregnancies becomes crucial. Preventive strategies include strict glycemic control in diabetic women, appropriate weight gain counseling, and systematic monitoring of fetal growth using both clinical and ultrasonographic methods. Ultrasonography, in particular, has emerged as the cornerstone of prenatal assessment of fetal growth. Traditional biometric parameters - biparietal diameter (BPD), head circumference (HC), abdominal circumference (AC), and femur length (FL) - are widely used to estimate fetal weight. Among these, abdominal circumference assumes particular importance in diabetic pregnancies, reflecting the characteristic insulin-mediated increase in fetal fat deposition [11].

Despite its advantages, ultrasonography is not without limitations. The accuracy of late third-trimester scans may be affected by several variables, including fetal position, maternal obesity, and reduced amniotic fluid volume. Moreover, the rapid fetal growth occurring during the final weeks of pregnancy reduces the predictive precision of estimated fetal weight (EFW), particularly in high-risk populations where growth trajectories may be irregular. In tertiary care facilities, where high-risk pregnancies are concentrated, the judicious application of ultrasonographic measurements assumes even greater relevance. Early and accurate identification of macrosomia facilitates individualized delivery planning, allowing obstetricians to weigh the risks of vaginal versus cesarean delivery and to ensure preparedness for potential complications such as shoulder dystocia or postpartum hemorrhage. This proactive approach contributes to improving both maternal and neonatal safety.

In summary, fetal macrosomia represents a multifactorial obstetric challenge with significant implications for maternal and neonatal health. Ultrasonography remains the primary non-invasive tool for its assessment, with continuous advancements enhancing its predictive accuracy. However, a nuanced understanding of its limitations, particularly in high-risk populations such as women with diabetes, is essential. The present study seeks to evaluate the role of ultrasonographic measurements in predicting macrosomia among fetuses of high-risk antenatal women in a tertiary care hospital setting. 

## Materials and methods

Study setting

This hospital-based study was conducted at a tertiary care centre catering to a large and diverse patient population in Chennai in South India. The hospital’s obstetrics and gynecology department is equipped with modern ultrasonographic facilities and specialized units for managing high-risk pregnancies, making it an appropriate setting for evaluating fetal macrosomia using advanced imaging parameters. On average, the department manages approximately 1,200-1,500 obstetric cases per month (around 15,000-18,000 annually), which supports the reproducibility and generalizability of the study findings to similar high-volume centres.

Study design

The research followed a prospective observational study design, which allowed systematic collection of data from antenatal women identified as high-risk. This design was chosen to prospectively follow the participants from 35-36 weeks of gestation until delivery, enabling real-time assessment of fetal biometric and ultrasonographic parameters and correlating them with birth outcomes. The study was carried out over a one-year period, spanning from July 2023 to June 2024. 

Sample size calculation

The required sample size for the study was determined based on the prevalence of macrosomia reported in a previous study, which was taken as 7.3% [12]. Using this prevalence, with a 95% confidence interval, 80% power, and an absolute precision of 5%, the estimated minimum sample size was approximately 115. Due to some loss to follow-ups, a total of 100 antenatal women were included in the present study.

Sampling methodology

A purposive sampling technique was employed. This non-probability sampling method was appropriate for the study as the focus was on a specific group of high-risk antenatal women rather than the general population. Women with particular risk determinants known to influence fetal growth and the development of macrosomia were selectively included. This approach maximized the likelihood of obtaining meaningful associations between ultrasonographic measurements and macrosomia.

Inclusion criteria

The study population consisted of antenatal women in their late third trimester, specifically between 35 and 36 weeks of gestation, who were identified as having one or more high-risk determinants. Women diagnosed with gestational diabetes mellitus (GDM) were included, given its well-established association with fetal overgrowth and macrosomia. In addition, women with anemia complicating pregnancy were enrolled, as this condition may indirectly influence maternal-fetal outcomes. Pregnant women with a history of epilepsy were also considered eligible, owing to the potential impact of both the disease and its pharmacological management on pregnancy outcomes. Similarly, those with cardiac disease or bronchial asthma during pregnancy were included, as these comorbidities can affect maternal-fetal physiology and may alter fetal growth patterns. Finally, women with hypertensive disorders of pregnancy, including gestational hypertension and pre-eclampsia, were included due to the increased obstetric risks associated with these conditions.

Exclusion criteria

To maintain study homogeneity and avoid confounding variables that could interfere with fetal growth assessment, specific exclusion criteria were applied. Multiple pregnancies were excluded, as twins and higher-order gestations carry unique growth trajectories and complication profiles. Pregnancies complicated by known fetal anomalies were also excluded since congenital malformations may independently influence fetal size, rendering ultrasonographic correlations less reliable. Additionally, women who were unwilling to participate or who did not provide informed consent were excluded from the study in adherence to ethical standards.

Data collection process

The data collection process was carefully structured to ensure methodological rigor and reliability. Eligible participants were first identified during routine antenatal visits conducted between 35 and 36 weeks of gestation. High-risk status was determined on the basis of detailed medical and obstetric history, supplemented by available clinical records. Once identified, each eligible woman was counselled about the study. The objectives, methodology, potential benefits, and possible risks were explained clearly in the participant’s preferred language. Following this discussion, written informed consent was obtained from all participants. A structured proforma was then used to systematically document demographic details like age, socioeconomic status based on Modified BG Prasad Scale [13], relevant obstetric history, high-risk factors, and baseline clinical parameters.

Ultrasonographic measurements

Following enrollment, each participant underwent targeted ultrasonographic evaluation at 35-36 weeks of gestation. Standardized protocols were followed to minimize inter-observer variability and enhance measurement accuracy. Umbilical cord thickness (UCT) was measured at a free loop of the cord using both transverse and longitudinal views, as increased UCT has been associated with excessive fetal growth and fat accumulation. Shoulder pad thickness (SPT) was measured at the level of the fetal shoulder girdle to identify disproportionate fat deposition, a hallmark feature of macrosomic infants, particularly those of diabetic mothers. Lastly, the fetal fat layer (FFL), typically measured at the anterior abdominal wall, was evaluated as an indirect indicator of insulin-mediated subcutaneous fat deposition. These three parameters, combined with standard biometry, were expected to provide a more comprehensive prediction of macrosomia [14].

Follow-up

All participants were followed up during their subsequent antenatal visits and continued to be monitored until delivery. Key obstetric outcomes such as birth weight, mode of delivery, and complications during labor were meticulously documented. Immediate neonatal outcomes, including events such as neonatal hypoglycemia and birth trauma, were also recorded. These outcome measures were then correlated with the ultrasonographic parameters obtained at 35-36 weeks to determine the predictive reliability of the measurements in identifying fetal macrosomia among high-risk pregnancies.

Ethical considerations

Ethical approval for the study was obtained from the Institutional Human Ethics Committee of Sri Balaji Medical College and Hospital (002/SBMCH/IHEC/2023/2042) prior to initiation. Written informed consent was obtained in the local language. Confidentiality of all participants was ensured by anonymizing identifiers during data collection and analysis. Sensitive issues such as maternal comorbidities and pregnancy outcomes were handled with utmost privacy and respect. Necessary permissions for the use of the questionnaire were obtained from the ethics committee.

Statistical analysis

The collected data were first entered into Microsoft Excel (Microsoft Corporation, Redmond, USA) and then analyzed using SPSS software version 26.0 (IBM Corp., Armonk, USA). Descriptive statistics, including mean, standard deviation, frequency, and percentage, were used to summarize the baseline characteristics of the study participants as well as the distribution of ultrasonographic parameters. Comparative analyses were performed using independent t-tests to compare continuous variables, while Chi-square/Fisher's exact tests were applied for categorical variables, including maternal comorbidities and mode of delivery. For all statistical tests, a p-value less than 0.05 was considered to indicate statistical significance.

## Results

The majority of antenatal women were aged 31-35 years (44%), followed by 26-30 years (26%), while only 1% were <20 years. More than half were primigravida (59%), with 27% being second gravida. In terms of socioeconomic status, 26% belonged to the upper class, and 25% to the lower middle class. With respect to nutritional status based on the WHO classification of BMI, overweight women formed the largest group (53%), while 16% were obese. The most common high-risk condition was gestational diabetes mellitus (65%), followed by anemia (16%) and hypertensive disorders (9%). Regarding the mode of delivery, more than half underwent lower segment caesarean section (LSCS)** **(57%), while 25% had a normal vaginal delivery. Among the newborns, 63% were male (Table 1).

**Table 1:** Sociodemographic characteristics of study participants (n=100) * Modified BG Prasad Scale [13].

Variable	Category	n	%
Age (years)	<20	1	1.0
	21–25	16	16.0
	26–30	26	26.0
	31–35	44	44.0
	>35	13	13.0
Parity	Primigravida	59	59.0
	G2	27	27.0
	≥G3	14	14.0
Socioeconomic Status*	Upper	26	26.0
	Upper Middle	19	19.0
	Lower Middle	25	25.0
	Upper Lower	13	13.0
	Lower	17	17.0
BMI	Normal	31	31.0
	Overweight	53	53.0
	Obese	16	16.0
High-Risk Cases	Gestational Diabetes Mellitus	65	65.0
	Epilepsy	1	1.0
	Anemia	16	16.0
	Hypertensive Disorders	9	9.0
	Bronchial Asthma	7	7.0
	Heart Disease	2	2.0
Mode of delivery	Normal Vaginal Delivery	25	25
	Instrumental	18	18
	Lower Segment Caesarean Section (LSCS)	57	57
Sex of the newborn	Boy	63	63
	Girl	37	37

Among the ultrasound parameters assessed, umbilical cord thickness (UCT) greater than the 90th percentile was observed in 45%, while the remaining 55% had UCT below this level. Fetal fat layer (FFL) >5 mm was detected in 49%, whereas 51% had FFL ≤5 mm. Similarly, shoulder pad thickness (SPT) >12 mm was present in 49%, compared to 51% with SPT ≤12 mm. An estimated fetal weight (EFW) >4.0 kg was also noted in 49%, while 51% had an EFW below this threshold (Table 2).

**Table 2:** Description of ultrasonographic parameters among the study participants (n=100)

Ultrasound Marker	Category	Number of Women (n)	Percentage (%)	Description
Umbilical Cord Thickness (UCT)	> 90th percentile	45	45%	Important anthropometric marker in high-risk pregnancies
	< 90th percentile	55	55%	
Fetal Fat Layer (FFL)	> 5 mm	49	49%	Marker of fetal soft tissue thickness
	< 5 mm	51	51%	
Shoulder Pad Thickness (SPT)	> 12 mm	49	49%	Sonographic parameter for fetal soft tissue development
	< 12 mm	51	51%	
Estimated Fetal Weight (EFW)	> 4.0 kg	49	49%	Critical for monitoring fetal growth
	< 4.0 kg	51	51%	

**Figure 1:**
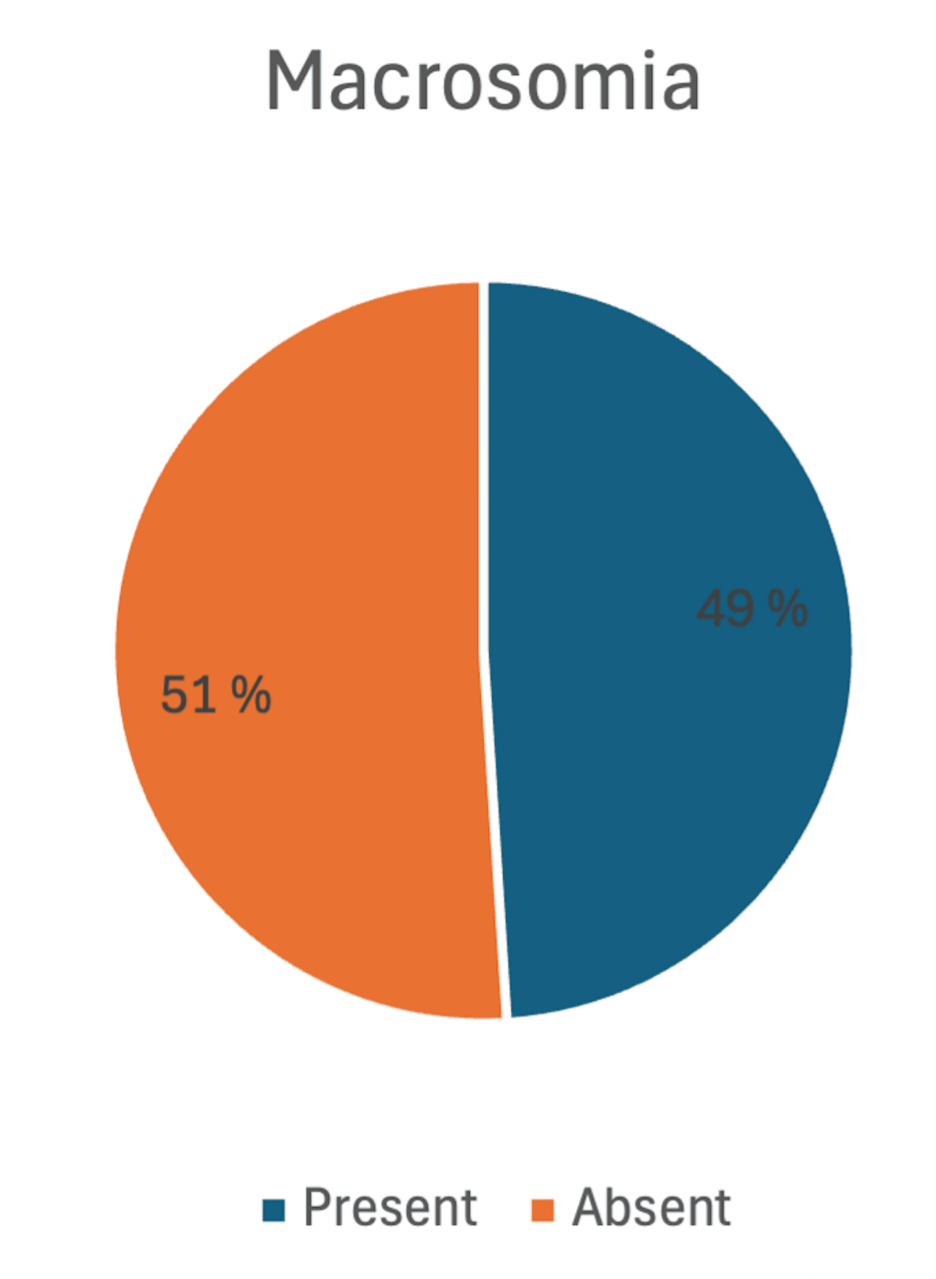
Prevalence of macrosomia among the study participants (n=100)

The prevalence of fetal macrosomia in this study was 49% (n=49/100), while 51% (n=51/100) of newborns were non-macrosomic. This indicates that nearly half of all high-risk pregnancies resulted in macrosomic births. The 95% confidence interval (CI) for the prevalence of macrosomia was 39.2%-58.8%, suggesting a precise estimate within a narrow range. These findings highlight the substantial burden of macrosomia among high-risk pregnancies in the studied population (Figure 1).

In the present study, the prevalence of macrosomia varied significantly across maternal and neonatal characteristics, as shown in Table 3. Women aged 31-35 years showed the highest prevalence (72.7%), while none of those aged <20 years had macrosomic babies (p<0.001). Primigravida mothers had a markedly higher prevalence (71.2%) compared to G2 (11.1%) and ≥G3 (28.6%) (p<0.001). Socioeconomic status was also strongly associated, with 92.3% of women from the upper class delivering macrosomic babies, whereas none from the upper-lower group had macrosomia (p<0.001). By BMI, macrosomia was most frequent in overweight women (75.5%), compared to only 12.9% in normal BMI (p<0.001). Among high-risk groups, GDM mothers had a prevalence of 75.4%, while no macrosomia was reported in women with anemia, hypertensive disorders, asthma, or heart disease (p<0.001). With respect to delivery mode, 95.0% of macrosomic babies were delivered by cesarean section, compared to only 15.4% by vaginal delivery (p<0.001). Finally, macrosomia was more common in newborn boys (57.1%) than girls (35.1%) (p=0.034). 

**Table 3:** Association of socio-clinical characteristics of pregnant women with macrosomia (N=100) *Modified BG Prasad Scale [11] ^#^Chi-square/Fisher's Exact test A p-value of less than 0.05 is statistically significant

Variable	Categories	Macrosomia Present n (%)	Macrosomia Absent n (%)	Test statistic (Chi^2^)#	P-value
Age (years)	<20	0 (0.0%)	1 (100.0%)	22.98	<0.001
	21-25	2 (12.5%)	14 (87.5%)		
	26-30	12 (46.2%)	14 (53.8%)		
	31-35	32 (72.7%)	12 (27.3%)		
	>35	3 (23.1%)	10 (76.9%)		
Parity	Primigravida (PRIMI)	42 (71.2%)	17 (28.8%)	29.47	<0.001
	G2	3 (11.1%)	24 (88.9%)		
	>= G3	4 (28.6%)	10 (71.4%)		
Socioeconomic status (SES)*	Upper	24 (92.3%)	2 (7.7%)	35.52	<0.001
	Upper Middle	10 (52.6%)	9 (47.4%)		
	Lower Middle	10 (40.0%)	15 (60.0%)		
	Upper Lower	0 (0.0%)	13 (100.0%)		
	Lower	5 (29.4%)	12 (70.6%)		
BMI Status	Normal	4 (12.9%)	27 (87.1%)	33.04	<0.001
	Overweight	40 (75.5%)	13 (24.5%)		
	Obese	5 (31.3%)	11 (68.8%)		
High-Risk Cases	Gestational Diabetes Mellitus (GDM)	49 (75.4%)	16 (24.6%)	51.73	<0.001
	Epilepsy	0 (0.0%)	1 (100.0%)		
	Anemia	0 (0.0%)	16 (100.0%)		
	Hypertensive Disorders of Pregnancy (HDP)	0 (0.0%)	9 (100.0%)		
	Bronchial Asthma (BA)	0 (0.0%)	7 (100.0%)		
	Heart Disease	0 (0.0%)	2 (100.0%)		
Mode of Delivery	Normal Vaginal (NVD)	2 (15.4%)	11 (84.6%)	33.52	<0.001
	Instrumental	9 (75.0%)	3 (25.0%)		
	Lower Segment Cesarean Section (LSCS)	38 (95.0%)	2 (5.0%)		
Sex of the newborn	Boy	36 (57.1%)	27 (42.9%)	3.68	0.034
	Girl	13 (35.1%)	24 (64.9%)		

In the present study, ultrasound markers showed a strong and statistically significant association with macrosomia, as shown in Table 4. All women with UCT >90th percentile, FFL >5 mm, SPT >12 mm, or EFW >4.0 kg had macrosomic babies (100%), while none without these markers developed macrosomia (p<0.001 for all). Conversely, macrosomia was absent in women with UCT <90th percentile (80%), FFL <5 mm (100%), SPT <12 mm (100%), and EFW <4.0 kg (100%). These findings highlight that UCT, FFL, SPT, and EFW are highly predictive sonographic markers of macrosomia.

**Table 4:** Association of ultrasound markers with macrosomia (n=100) ^#^Chi-square/Fisher's Exact test A p-value of less than 0.05 is statistically significant UCT: umbilical cord thickness; FFL: fetal fat layer; SPT: shoulder pad thickness; EFW: estimated fetal weight

Parameter	Macrosomia Present n (%)	Macrosomia Absent n (%)	Test statistic (Chi^2^)#	P-value
UCT >90%	45 (100.0%)	0 (0.0%)	45.0	<0.001
UCT <90%	4 (20.0%)	16 (80.0%)		
FFL >5mm	49 (100.0%)	0 (0.0%)	65.3	<0.001
FFL <5mm	0 (0.0%)	16 (100.0%)		
SPT >12mm	49 (100.0%)	0 (0.0%)	65.3	<0.001
SPT <12mm	0 (0.0%)	16 (100.0%)		
EFW >4kg	49 (100.0%)	0 (0.0%)	65.3	<0.001
EFW <4kg	0 (0.0%)	16 (100.0%)		

## Discussion

This study examined the associations between maternal comorbidities, sociodemographic factors, and novel ultrasonographic parameters in predicting fetal macrosomia among high-risk pregnancies. The findings underscore a powerful and consistent relationship between specific ultrasound parameters - Umbilical Cord Thickness (UCT), Fetal Fat Layer (FFL), and Shoulder Pad Thickness (SPT) - and the diagnosis of Gestational Diabetes Mellitus (GDM) and the subsequent development of macrosomia. 

GDM was predominantly observed in primigravida women (74.6%), which aligns with findings by Swaminathan et al. and Chakraborty et al., who attribute this to urban lifestyle factors, delayed childbearing, and elevated BMI among first-time mothers [15,16]. Conversely, anemia showed an inverse relationship with parity, being most prevalent in multiparous women (50%), consistent with the iron-depletion hypothesis highlighted by Cohen et al. (2006) [17]. Hypertensive disorders were concentrated in early pregnancies, reinforcing the theory of immunological maladaptation as suggested by Cohen et al. [17]. Bronchial asthma increased with parity, while epilepsy and cardiac complications were observed exclusively in parity-one women.

Beyond ultrasonography, the study identifies key demographic and maternal health factors strongly correlated with macrosomia. The very high rates of macrosomia among women of advanced maternal age (31-35 years), primigravidas, those from an upper socioeconomic class, and particularly those who are overweight (75.5%), paint a clear picture of the metabolic and nutritional determinants of fetal overgrowth, aligning with previous literature [18,19]. The overwhelming association of GDM with macrosomia (75.4%) reaffirms its position as the primary medical driver of this condition [20]. Furthermore, the mode of delivery data strongly aligns with clinical expectations, demonstrating that macrosomia significantly increases the likelihood of a cesarean section, likely due to concerns about cephalopelvic disproportion and associated risks of obstructed labor and shoulder dystocia.

The near-perfect concordance between ultrasonographically estimated fetal weight (EFW) and actual birth weight in diagnosing macrosomia is a critical finding. The fact that all fetuses with an EFW >4.0kg were macrosomic and none below this threshold were classified as such indicates a high degree of diagnostic accuracy within this study population [21]. This validates the use of ultrasound as a reliable tool for antenatal diagnosis and planning for macrosomic infants, which is essential for optimizing delivery management and preparing for potential neonatal complications.

Delivery outcomes in the present study further reinforced the significance of these risk factors. A majority of high-risk women required cesarean section (57%), with macrosomia being a major determinant of operative intervention. Macrosomic births were strongly associated with cesarean and instrumental deliveries (p < 0.001), corroborating global evidence linking GDM and macrosomia with higher surgical delivery rates [22]. Male infants were significantly more likely to be macrosomic compared to females (p = 0.034), while maternal age between 31 and 35 years posed the greatest risk for macrosomia, reflecting demographic and metabolic vulnerabilities. Moreover, Villar et al. (2015), in the INTERGROWTH-21st study, demonstrated consistently higher birth weights among males across various ethnic populations, supporting the global relevance of these findings. Recognition of this sex-based growth differential may aid clinicians in refining fetal growth assessments and stratifying risk for delivery complications [23].

However, several limitations must be considered when interpreting these results. The sample size, particularly for certain sub-groups, is considerably small. As a single-center study, the results may be influenced by local population characteristics and clinical practices. The study design does not account for all potential confounding variables, such as the specific level of glycemic control in women with GDM, which is a crucial factor influencing fetal growth. Finally, the thresholds used for UCT, FFL, and SPT, while significant in this study, require validation in larger, multi-center cohorts to establish standardized cut-off values for widespread clinical use.

The implications of this study are twofold. For clinical practice, it strongly advocates for the incorporation of detailed soft-tissue ultrasonography - specifically measuring UCT, FFL, and SPT - into the antenatal surveillance of high-risk women, especially those with GDM or significant risk factors like obesity. These markers could enhance the predictive value of EFW alone, allowing for earlier and more precise identification of macrosomia, which in turn facilitates better-informed decisions regarding the timing and mode of delivery to improve maternal and neonatal outcomes. For research, these findings highlight the need for prospective longitudinal studies to establish causal relationships and define universal diagnostic thresholds for these sonographic markers. Future work should also focus on integrating these measurements with biochemical markers of glycemic control to develop a comprehensive predictive model for fetal macrosomia.

## Conclusions

By demonstrating the predictive value of these ultrasonographic markers, the study advocates their routine integration into high-risk pregnancy screening protocols, particularly for women with GDM or elevated BMI. Such proactive monitoring can facilitate timely interventions, optimize delivery planning, and ultimately reduce maternal and neonatal morbidity and mortality associated with macrosomia. These findings support a shift toward evidence-based, ultrasound-guided antenatal care strategies aimed at improving outcomes for both mother and child. Proactive identification of macrosomia risk allows obstetricians to anticipate complications such as shoulder dystocia, birth trauma, or operative delivery, and to plan timely interventions, including dietary modification, glycemic control, and decisions regarding mode and timing of delivery. Such measures not only enhance maternal safety but also reduce the burden of neonatal morbidity, including hypoglycemia, respiratory distress, and long-term metabolic complications.
